# Body mass, temperature, and pathogen intensity differentially affect critical thermal maxima and their population‐level variation in a solitary bee

**DOI:** 10.1002/ece3.10945

**Published:** 2024-02-15

**Authors:** Laura J. Jones, Douglas A. Miller, Rudolf J. Schilder, Margarita M. López‐Uribe

**Affiliations:** ^1^ Intercollege Graduate Degree Program in Ecology The Pennsylvania State University University Park Pennsylvania USA; ^2^ Department of Entomology, Center for Pollinator Research The Pennsylvania State University University Park Pennsylvania USA; ^3^ Earth and Environmental Systems Institute The Pennsylvania State University University Park Pennsylvania USA; ^4^ Department of Biology The Pennsylvania State University University Park Pennsylvania USA

**Keywords:** climate, critical thermal maxima, heat tolerance, parasite, sex

## Abstract

Climate change presents a major threat to species distribution and persistence. Understanding what abiotic or biotic factors influence the thermal tolerances of natural populations is critical to assessing their vulnerability under rapidly changing thermal regimes. This study evaluates how body mass, local climate, and pathogen intensity influence heat tolerance and its population‐level variation (SD) among individuals of the solitary bee *Xenoglossa pruinosa*. We assess the sex‐specific relationships between these factors and heat tolerance given the differences in size between sexes and the ground‐nesting behavior of the females. We collected *X*. *pruinosa* individuals from 14 sites across Pennsylvania, USA, that varied in mean temperature, precipitation, and soil texture. We measured the critical thermal maxima (CT_max_) of *X*. *pruinosa* individuals as our proxy for heat tolerance and used quantitative PCR to determine relative intensities of three parasite groups—trypanosomes, *Spiroplasma apis* (mollicute bacteria), and *Vairimorpha apis* (microsporidian). While there was no difference in CT_max_ between the sexes, we found that CT_max_ increased significantly with body mass and that this relationship was stronger for males than for females. Air temperature, precipitation, and soil texture did not predict mean CT_max_ for either sex. However, population‐level variation in CT_max_ was strongly and negatively correlated with air temperature, which suggests that temperature is acting as an environmental filter. Of the parasites screened, only trypanosome intensity correlated with heat tolerance. Specifically, trypanosome intensity negatively correlated with the CT_max_ of female *X*. *pruinosa* but not males. Our results highlight the importance of considering size, sex, and infection status when evaluating thermal tolerance traits. Importantly, this study reveals the need to evaluate trends in the variation of heat tolerance within and between populations and consider implications of reduced variation in heat tolerance for the persistence of ectotherms in future climate conditions.

## Graphical abstract

This study evaluates how body mass, local climate, and pathogen intensity influence heat tolerance and its population‐level variation among individuals of the solitary bee *Xenoglossa pruinosa*. We did not find evidence of microclimate predicting mean heat tolerance in *X*. *pruinosa*, but we found that the population‐level standard deviation in heat tolerance (CT_max_) decreased by 0.72°C with every 1°C increase in maximum temperature. In addition, trypanosome parasite intensity negatively correlated with the CT_max_ of females, but not males. This study reveals that temperature may act as an environmental filter, and exemplifies the need to evaluate trends in heat tolerance variation within and between populations.

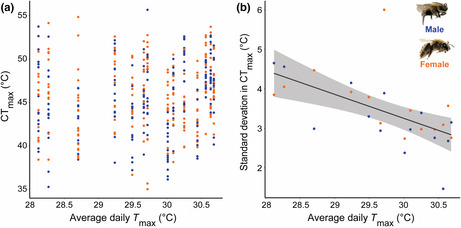

## 1 INTRODUCTION

Understanding how organisms tolerate temperature extremes is critical for assessing the threat of climate change on species' distributions and persistence (Buckley & Huey, 2016; Schulte, 2015). Small‐bodied ectotherms are considered a group highly vulnerable to changing climate regimes, as their capacity to maintain body temperatures essential for cellular and bodily processes is dependent on external conditions (Gunderson & Stillman, 2015; Harvey et al., 2020; Kingsolver et al., 2013; Sinclair et al., 2016). There is growing interest in evaluating the heat tolerance, acclimation capacity, and plasticity of these traits in ectotherms as they relate to changing abiotic conditions like ambient temperature (Barley et al., 2021; Layne et al., 1987; Overgaard et al., 2011; Oyen & Dillon, 2018; Shah et al., 2017; Sobek et al., 2011). However, few studies characterize biotic impacts on thermal tolerance in natural populations in addition to abiotic factors, such as pathogen infection (Hector et al., 2021). Additionally, traits such as body size or fat content can impact how organisms tolerate temperature (González‐del‐Pliego et al., 2020; Oyen et al., 2016; Ribeiro et al., 2012; Roeder et al., 2021). Thus, it is vital to consider individual condition, as well as the abiotic and biotic factors that individuals experience in natural environments, when evaluating the heat tolerance of populations in order to predict their success in future thermal regimes.

Heat tolerance is a critical measurement for understanding terrestrial ectotherms' distributions given that these species have a limited capacity to adjust their upper thermal tolerance via adaptive processes (i.e., Brett's heat‐invariant hypothesis; Brett, 1956) and are sensitive to climate conditions at small spatial scales (Montejo‐Kovacevich et al., 2020; Roeder et al., 2021). Despite its importance, there is only limited evidence of plasticity in heat tolerance and acclimation to warmer conditions for terrestrial ectotherms (Cavieres et al., 2019; Sobek et al., 2011); however, wide population‐level variation is often seen in heat tolerance assays. These findings support the need to increase our characterization of heat tolerance among ectotherms to understand which species are more restricted by their upper thermal tolerance and to relate the heat tolerance of populations and variation among individuals to the environmental conditions they experience. Currently, most studies focus on relating thermal tolerance to air temperature or elevational and latitudinal gradients (Angilletta, 2009; Baudier et al., 2018; Sunday et al., 2014). However, small‐bodied ectotherms can experience vastly different microclimates in relatively small areas regardless of air temperature depending on the specific habitats that they occupy (Herrera, 1997). For example, many ant species build nests underground where they are largely buffered from extreme daily air temperature variation (Baudier et al., 2015). Instead, the thermal environment underground depends largely on soil texture, with sandier soils having less stable temperature conditions due to lower thermal inertia and reduced capacity to hold water (Campbell & Norman, 1998). Thus, when evaluating the heat tolerance of species in their natural environments, it is important to consider what abiotic factors are the most relevant to assess the daily temperatures they are exposed to.

In addition to climate conditions experienced at different spatial scales, both behavioral and physical traits may also influence the response of ectotherms to their thermal environment. Terrestrial ectotherms will show preferences for, and seek out, thermal environments within their thermal tolerance range to reduce time in stressful conditions (Ma et al., 2018; Ng et al., 2017; Nielsen & Papaj, 2017; Rangel‐Patiño et al., 2020). However, organisms often travel outside of their preferred thermal environments and experience heat stress when foraging (Youngsteadt et al., 2023). Physical traits, like body size, can influence the upper thermal conditions that organisms tolerate outside of their preferred temperature ranges. For example, in many terrestrial ectotherms including frogs and insects, larger species or individuals within a species have higher heat tolerances (Baudier et al., 2015; Ribeiro et al., 2012; von May et al., 2019), which has been attributed to reduced rates of water loss. However, this trend does not hold true across all studies (e.g., this relationship is not found in all bee studies; Gonzalez et al., 2020; Hamblin et al., 2017; Oyen et al., 2016; Oyen & Dillon, 2018). While the impact of size on heat tolerance across species is variable, known differences in size or behavior between sexes may explain within‐species variation. For example, female invertebrates are often larger than males and invest more in critical behavior like parental care or food provisioning (Shine, 1989; Wong et al., 2013), which may suggest higher female heat tolerance. However, few studies have evaluated sex differences in heat tolerance traits (Laidlaw et al., 2020; Madeira et al., 2012; Mitchell & Hoffmann, 2010). Given the known physical and behavioral differences between sexes for many species, comparing their heat tolerance may be an essential next step for inferring the threat of climate change on the future of a species.

Finally, a challenge that emerges in assessing the heat tolerance of ectotherms collected from natural environments is the uncertainty in their current condition, such as infection status. Climate change is expected to increase rates of parasitism due to novel interactions between parasites and vulnerable hosts resulting from range shifts and heat stress (Altizer et al., 2013; Gallana et al., 2013). Pathogen infection has been shown to reduce thermal tolerance in frogs infected with the fungus *Batrachochytrium dendrobatidis* (Greenspan et al., 2017), trout infected with the myxozoan *Tetracapsuloides bryosalmonae* (Bruneaux et al., 2017), and spider mites infected with the bacteria *Wolbachia* or *Spiroplasma* (Zhu et al., 2021), among others. In contrast, some infections have been shown to increase thermal tolerance (e.g., aphids infected with barley yellow dwarf virus or whiteflies infected with the bacteria *Cardinium*; Porras et al., 2020; Yang et al., 2021). Despite these strong implications of parasite infection for thermal tolerance, studies of natural populations often do not screen individuals for potential parasitism and its effects. Including parasite screening of individuals collected from natural populations may help explain some of the wide variation seen in heat tolerance assays.

Here, we evaluate how abiotic and biotic factors influence the heat tolerance of hoary squash bee (*Xenoglossa pruinosa*, formerly *Eucera* (*Peponapis*) *pruinosa*) populations in Pennsylvania (USA) (Freitas et al., 2023). This is a solitary species that exhibits sex differences in physiology and behavior. Females are larger than males, provision pollen for offspring in the morning through midday, and nest underground, which buffers them from high air temperatures and variation in temperature (Danforth et al., 2019; Hurd et al., 1974). Soil texture may impact the degree of thermal buffering offered to female *X*. *pruinosa* in their ground nests because sandy soils have a lower heat capacity. In contrast, males are smaller, only forage for nectar, and buffer themselves from heat by retreating into wilted flowers as protection after foraging concludes midday. So, males are exposed to more variable ambient temperature conditions than females during the day and night. Previously, we have found high prevalences of common bee parasite groups—trypanosomes (e.g., *Crithidia mellificae*), microsporidia (*Vairimorpha apis* formerly known as *Nosema apis*), and bacteria (*Spiroplasma apis*)—in *X*. *pruinosa* collected at these sites (Jones et al., 2022). These parasites are known to elicit disease symptoms that may affect water content or energy allocation in their hosts, including dysentery (i.e., water loss) and impaired cognition (i.e., reduced foraging), in other bee species (Figueroa et al., 2019; Gegear et al., 2006; Graystock et al., 2013; McArt et al., 2014; Schwarz et al., 2014). We hypothesize that (1) heat tolerance increases with body size, (2) male heat tolerance increases with ambient temperatures above ground whereas female heat tolerance increases with sandier soils, and (3) parasite infection reduces heat tolerance in *X*. *pruinosa*. To investigate our hypotheses, we determined the critical thermal maxima (CT_max_) of wild‐caught *X*. *pruinosa* that varied in body size across a thermally variant geographic range. We determined the relative quantities of parasites in each individual. Lastly, we characterized temperature, precipitation, and soil texture at each site.

## 2 MATERIALS AND METHODS

### 2.1 Study system and collections

**FIGURE 1:**
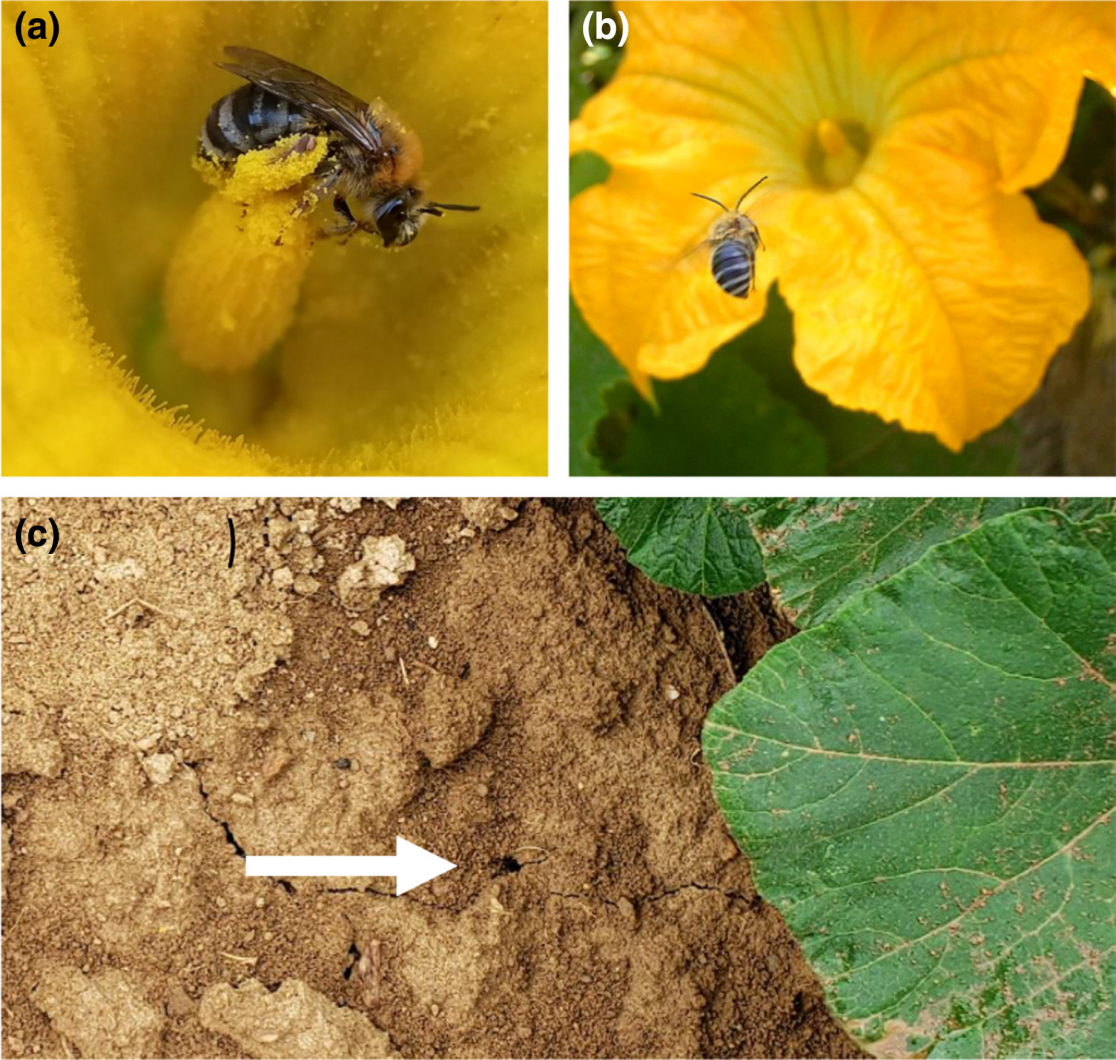
Study organism *Xenoglossa pruinosa*. (a) Female *X*. *pruinosa* provisioning pollen for her offspring (see scopa on hind leg packed with pollen). (b) Male *X*. *pruinosa* in flight to a *Cucurbita* flower. (c) The white arrow indicates a female *X*. *pruinosa* nest partially shaded by *Cucurbita* leaves. Photography by Laura J. Jones.

*Xenoglossa pruinosa* is a solitary, ground‐nesting species that exclusively feeds on pollen from plants in the genus *Cucurbita* (pumpkin, squash, and gourds) (Figure 1). This bee recently expanded its geographic range northward from Mexico and the southwestern United States to the province of Quebec over the past ~2–3 kya, following the human cultivation of its host plants (López‐Uribe et al., 2016; Pope et al., 2023). Across their range, *X*. *pruinosa* closely track the flowering phenology of *Cucurbita*, and are therefore active aboveground as adults during the months of July and August in Pennsylvania (USA). We collected female and male *X*. *pruinosa* (*n* = 15 ± 2.14 SD individuals per sex per site) from 14 sites (>1 km apart) across Pennsylvania (USA) from July 18th to August 2nd of 2019 (Figure S1; raw site data file available at https://doi.org/10.5061/dryad.hhmgqnkp9). The *Cucurbita* fields were 3.17 ± 2.36 hectares in size. Individuals were captured directly from *Cucurbita* flowers and were examined for sex and species identification. Individuals were placed on ice to induce chill coma during travel to the laboratory from the field site. This was done to reduce variation in energy and water loss due to activity or stress during transport. Exposing bee individuals to cold stress before CT_max_ assays has been demonstrated to have no effect on CT_max_ outcomes (see Gonzalez, Oyen, Aguilar, et al., 2022; Gonzalez, Oyen, Ávila, & Ospina, 2022; Oyen & Dillon, 2018), and we are only comparing CT_max_ between the individuals in this study. The time spent in chill coma varied between 30 min and 4 h due to distance to The Pennsylvania State University, Centre Co. PA (USA) (Figure S1). We included time spent in chill coma as a separate predictor in our models, though we found that it did not have an effect on CT_max_ (see Tables 1 and 2, Table S2). We also found that the ambient temperature at sites and time in chill coma do not correlate (*F* value = 1.84; *df* = 1/416; *p* = .18).

### 2.2 CT_max_ assays

We assessed the critical thermal maxima (CT_max_) of *X*. *pruinosa* individuals to evaluate how body mass, sex, and abiotic and biotic factors influence their heat tolerance. This measure indicates the upper physiological limit of an organism to perform under acute heat stress in controlled conditions and does not represent thermal preference (Angilletta, 2009). Two temperature ramping rigs were constructed, modeled after the set‐up described in Oyen and Dillon (2018). A proportional–integral–derivative (PID) controller was used to program the temperature ramping rate. The temperature ramping rate used was +0.25°C per minute, starting at 23°C. Each trial included two individuals in the control group that were not exposed to ramping temperature conditions to monitor for changes in condition caused by external factors. Individuals were weighed before and after CT_max_ experiments to account for the effect of mass, and were randomly assigned to the experimental or control group. All vials in the experimental group were set at a depth of 0.635 cm in the aluminum well plate. Pico Technology TC‐08 data loggers were used to record and track temperature from T‐type thermocouples sitting against the outside of each vial wall. The control group was kept at 23°C for the duration of the experiment and showed no change in condition during experiments. All vials were covered with perforated plastic wrap to allow for gas exchange, and vial walls were minimally coated in mineral oil to reduce climbing efforts. Any individual that did not recover from chill coma after 30 min at 23°C before the experiment was not used. We approximated CT_max_ as the temperature of the vial at which an individual lost neuromuscular control (e.g., uncontrolled muscle spasms). Individuals were removed from the temperature ramping rig after reaching CT_max_, weighed, and were stored at −80°C for parasite screening. We collected CT_max_ for 220 female *X*. *pruinosa* and 200 male *X*. *pruinosa*; however, two male *X*. *pruinosa* (sample IDs epl0331 and epl0647) were considered to be in poor condition and removed from analyses.

### 2.3 Environmental data collection

We collected temperature and precipitation data at each site to evaluate the impact of local climate on the heat tolerance of *X*. *pruinosa*. We included precipitation in our models because of its known negative association with both air temperature and solar irradiance due to overcast conditions in the summer (Trenberth & Shea, 2005). We collected daily minimum and maximum temperatures for the months of July and August during the collection period in 2019 from the model PestWatch (2.5 km grid; http://www.pestwatch.psu.edu/). We used the average daily maximum temperature for the month of July from PestWatch for analyses because most of our samples were collected in July. To validate the temperature data from this model, we collected temperature data from HOBO 8K Pendant data loggers placed in the field. We found that the data were correlated (Figure S2; raw site data file and R file available at https://doi.org/10.5061/dryad.hhmgqnkp9) (see Appendix for extended methods). We did not use the daily maximum temperature collected from the HOBO temperature loggers because the maximum temperatures measured appear to be affected by solar radiation exposure (see Figure S2). We collected average daily precipitation from the PRISM climate dataset (4 km grid; https://prism.oregonstate.edu) for the month of July for each site. The average daily maximum temperature in July was 29.7 ± 0.85°C across sites (Figures S1 and S2), and the average daily precipitation was 4.6 ± 1.5 mm.

We tested if female CT_max_ was predicted by soil texture due to their ground‐nesting behavior and the known association between soil texture and soil temperature (Campbell & Norman, 1998). To determine soil texture, we collected two soil samples (one at the edge of the field, one 11 meters into the field) from each site during September 2019. We used a soil core sampler (5 cm wide) and collected soil to a depth of 30.5 cm. Samples were pooled and sent to the Penn State Agricultural Analytical Services Lab for particle size testing. We confirmed nesting in the soils collected at two of our sites. The average proportions were 25 ± 16.6% sand, 46.3 ± 8.5% silt, and 28.5 ± 9.7% clay across sites.

### 2.4 Parasite selection, screening, and relative quantification

We screened *X*. *pruinosa* for trypanosomes (e.g., *Crithidia mellificae* and three unidentified trypanosomes most closely related to *Crithidia brevicula*, *Leptomonas podlipaevi*, and *Leptomonas pyrrhocoris*), *Spiroplasma apis* (mollicute bacteria), and *Vairimorpha apis* (microsporidian, formerly *Nosema apis*) to assess if parasite intensity (relative amount of a parasite in a sample compared to the base sample with the largest ΔC_T_, considering only positive samples) impacts CT_max_ (Jones et al., 2022). These parasites were selected because they are common in bees and are known to elicit sublethal symptoms, including dysentery, impaired foraging, and reduced colony‐founding success, in two other bee genera that visit *Cucurbita* farms in Pennsylvania (USA), *Apis* and *Bombus* (Figueroa et al., 2019; Gegear et al., 2006; Graystock et al., 2013; McArt et al., 2014; Schwarz et al., 2014). All three parasite groups are detected in the intestinal epithelium, but *S*. *apis* is additionally detected in the hemolymph (Meeus et al., 2012) and *Vairimorpha* spp. (*V*. *ceranae*) have been detected in the Malpighian tubules, fat bodies, and other tissues (Chen et al., 2009). These tissues and the hemolymph are critical for nutrient and waste transport, excretion, and energy storage, among other functions. While trypanosomes colonize intestinal tissue, these parasites have been found to impact the reproductive system as well, which may suggest that trypanosome infection induces changes in energy allocation (Brown et al., 2000; Goulson et al., 2018). All three parasite groups have been described at high prevalence at these sites (Jones et al., 2022).

Individual *X*. *pruinosa* were screened for parasites using qPCR as described in Jones et al. (2021). We dissected whole abdomens of *X*. *pruinosa* using sterilized equipment and extracted DNA using the E.Z.N.A.® Tissue DNA Kit (SKU: D3396; Omega Bio‐tek, Inc.). DNA was eluted in Diethyl Pyrocarbonate (DEPC)‐treated water. We quantified DNA concentration using a SpectraMax iD3 Multi‐Mode Microplate Reader (Molecular Devices, San Jose, CA, USA). We used previously designed primer pairs for all parasites and the 28S rRNA reference gene (Table S1). We included a negative control for each target gene and ran all reactions (40 ng DNA per reaction) and negative controls in triplicate. We considered a reaction negative if it did not amplify, if the qPCR machine determined two of the three replicates were inconclusive, or if the average cycle threshold (*C*_*T*_) exceeded 35 (Jones et al., 2021). We used the 2^−ΔΔCT^ method to calculate relative intensity separately per target gene (raw individual data file available at https://doi.org/10.5061/dryad.hhmgqnkp9) (Livak & Schmittgen, 2001). We selected the sample with the greatest Δ*C*_*T*_ (target *C*_*T*_ − reference *C*_*T*_) per target gene to be the base sample for that parasite. The *C*_*T*_ value of each target gene was kept in ratio with the *C*_*T*_ value for the 28S rRNA reference gene and normalized by the base sample.

### 2.5 Statistical analyses

All statistical analyses were conducted in R (v.4.1.2) (R Core Team, 2023) (see Appendix for extended methods, R code is provided at https://doi.org/10.5061/dryad.hhmgqnkp9). We fit linear mixed models (LMMs) to test the effects of our abiotic (temperature, precipitation, and soil texture) and biotic (parasite relative intensity) predictors on CT_max_ (function “lmer,” package lme4 (v.1.1‐23)) (Bates et al., 2015). We found that the proportions of sand, silt, and clay were correlated, so we conducted principle component analysis (PCA) to create a composite variable (principle component 1) to use as our proxy for soil texture in analyses (function “prcomp”; package stats (v.3.6.2)) (Becker et al., 1988). We controlled for the time that individuals spent in chill coma by including it as a fixed effect and site (*n* = 14) was included as a random effect. The first set of LMMs tested the effects of sex, body mass (measured before CT_max_ assays), temperature, precipitation, and soil texture on *X*. *pruinosa* CT_max_ (Table 1a,b and Table S2j) and on the standard deviation of CT_max_ within populations (Table 1c). We use standard deviation as our measure for CT_max_ variation to describe and compare within‐population variation in CT_max_. The second set of LMMs tested the effects of sex, starting body mass, and either trypanosome (Table 2d,e and Table S2k), *S*. *apis* (Table 2f,g and Table S2l), or *V*. *apis* (Table 2h,i and Table S2m) intensity on *X*. *pruinosa* CT_max_. We fit separate linear mixed models (LMMs) to test the effects of our abiotic and biotic predictors on CT_max_ because the latter set of LMMs used different subsets of samples to include only individuals that the parasite was detected in (intensity data). We used ANOVA to test significance (function “Anova”, Type II Wald F tests with degrees of freedom calculated using the Kenward‐Roger method; package car (v.3.0‐10)) (Fox & Weisburg, 2019) and used post‐hoc tests with *p*‐values adjusted using the Tukey method to compare CT_max_ between the sexes (function “emmeans”; package emmeans (v.1.5.1)) (Lenth et al., 2020). We fit a linear model (LM) to investigate the relationship between *S*. *apis* intensity and CT_max_ (Table S2l) which did not include a random effect due to overfitting (function “lm,” stats (v.4.1.2)). Models in Table S2 (j–m) included an interaction between sex and body mass. Standardized coefficients (*β*_std_) were calculated by scaling the coefficients (*β*) by the ratio of the standard deviation of the predictor over the standard deviation of the response (*β*_std_ = *β* × (SD_predictor_/SD_response_)). We calculated Moran's *I* to describe the spatial structure in CT_max_ and all parasite intensities, but found no evidence of spatial autocorrelation in any variables (Table S3) (function “Moran.I”, package ape (v.5.7‐1)) (Paradis & Schliep, 2019).

## 3 RESULTS

**FIGURE 2:**
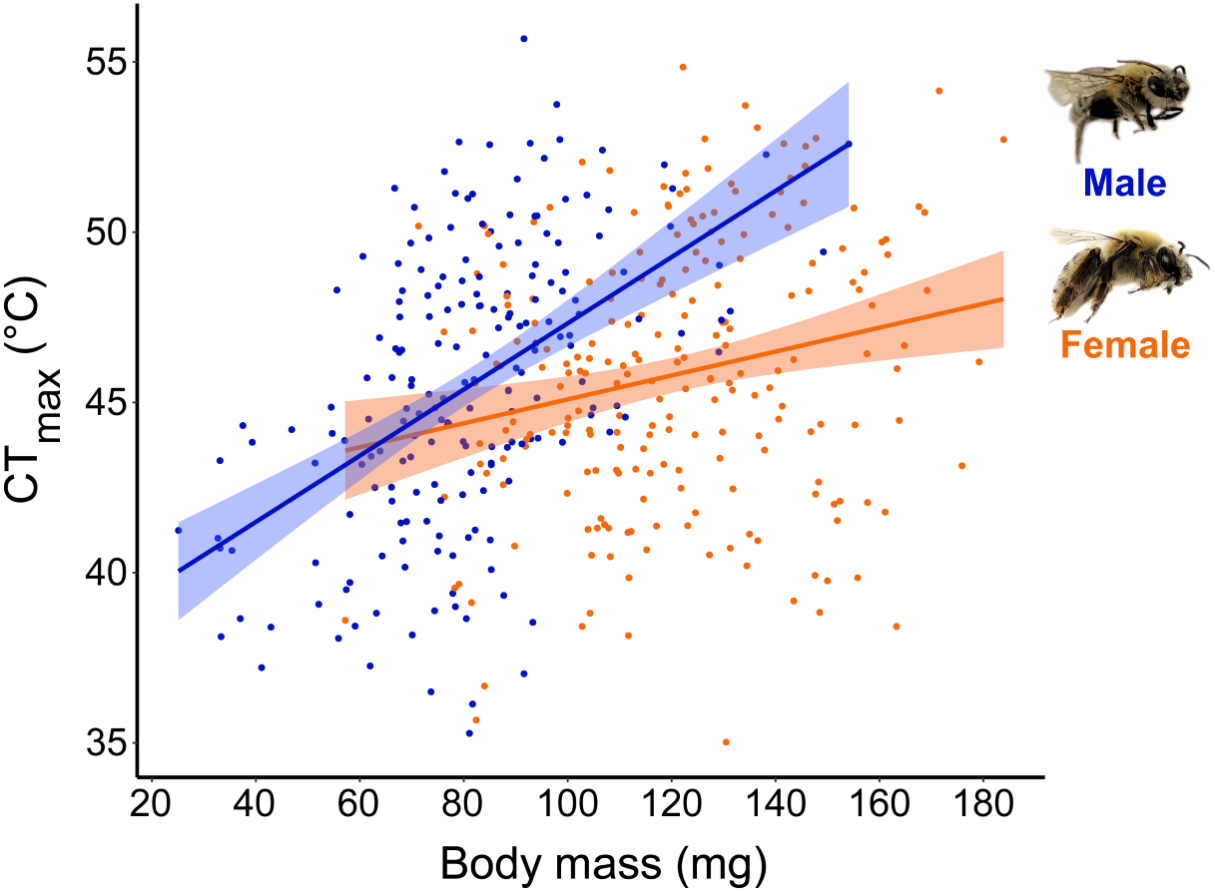
The effect of body mass on male (blue) and female (orange) *Xenoglossa pruinosa* CT_max_. Male (*y* = 0.097*x* + 37.6; 95% CI = (0.07, 0.12); *n* = 198) and female (*y* = 0.035*x* + 41.6; 95% CI = (0.01, 0.06); *n* = 220) *X*. *pruinosa* CT_max_ were positively correlated with body mass. The shaded regions around regression lines represent the 95% confidence intervals.

We found that *X*. *pruinosa* (females = 220; males = 198) captured from 14 *Cucurbita* sites across Pennsylvania (USA) had an average CT_max_ of 45.66 ± 4.03 SD °C (Figure 2 and Figure S3). Our models indicated an effect of sex on CT_max_; however, this effect is due to the interaction with body mass (Tables 1, 2 and Table S2). We employed Tukey test to compare CT_max_ between sexes and found no difference when mass was not considered (est. = 0.59, *t* ratio = 1.6, *p* = .11). Female CT_max_ was on average 45.82 ± 3.93°C and male CT_max_ was on average 45.47 ± 4.14°C (Figure S3). Overall, time spent in chill coma did not impact *E*. *pruinosa* CT_max_ in any models (Tables 1, 2 and Table S2).

### 3.1 Effect of body mass on CT_max_

We found that while CT_max_ was generally correlated with body mass, males had a stronger relationship between CT_max_ and body mass (coef. = 0.08, *p* < .0001) than females (coef. = 0.04, *p* = .0015) across our models (Tables 1, 2 and Table S2). Overall, males show twice the increase in degrees Celsius CT_max_ per milligram body mass compared to females (Figure 2; Table 1). However, body mass was not a significant predictor of CT_max_ in the model relating female *X*. *pruinosa* CT_max_ to *S*. *apis* intensity (*n* = 77/220 females, *p* = .65) (Table 2f).

### 3.2 Effects of temperature, precipitation, and soil texture on CT_max_

**FIGURE 3:**
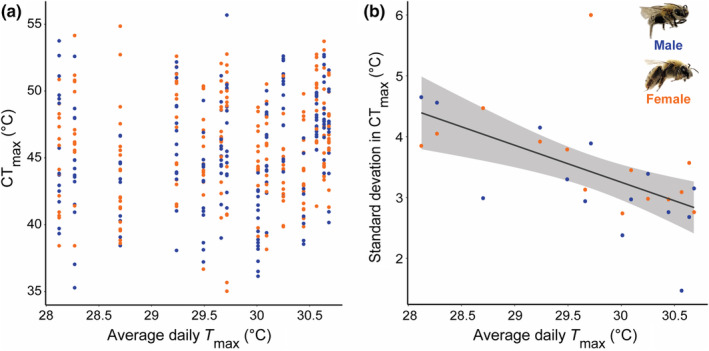
The effect of temperature (*T*_max_) on (a) *Xenoglossa pruinosa* CT_max_ and (b) population‐level variation (standard deviation) in *X*. *pruinosa* CT_max_. Average daily *T*_max_ is the average daily maximum temperature collected during the month of July (2019). (a) CT_max_ did not vary by average daily *T*_max_ (*n* = 418). (b) The standard deviation in CT_max_ was negatively correlated with average daily *T*_max_ (*y* = −0.61*x* + 21.5; 95% CI = (−0.95, −0.27); *n* = 28), and the relationship did not differ between male (blue) and female (orange) *X*. *pruinosa*. The shaded region around the regression line represents the 95% confidence interval.

We found that temperature (average daily *T*_max_), precipitation, and soil texture at *Cucurbita* farms did not predict *X*. *pruinosa* CT_max_ (Figure 3a; Table 1a,b and Table S2). However, we found a strong negative effect of *T*_max_ on the variation (SD) of CT_max_ found across sites (Figure 3b; Table 1c and Table S2). Overall, population‐level variation in CT_max_ decreased by 0.72°C per degree Celsius increase of average daily *T*_max_ (*p* = .01).

**TABLE 1:** Analysis of Variance (ANOVA) reporting Type II Wald *F* tests for linear mixed models (LMMs) including abiotic (environmental) predictors.

Mod.	*R* ^2^ (adj.)	*n*	Response	Predictor	Coef.	*β*_std_	*F* statistic	*df*	*p*‐Value
**a**	.06	220	Female CT_max_	**Body mass (mg)**	0.04	.22	10.353	1/212	.0015
				Average *T*_max_ (°C)	0.15	.03	0.061	1/9	.8108
				Average precipitation (mm)	0.36	.13	0.575	1/9	.4678
				Soil texture (PC1)	−0.10	−.04	0.064	1/9	.8065
				Time in chill coma (min)	−0.01	−.13	0.673	1/9	.4331
**b**	.2	198	Male CT_max_	**Body mass (mg)**	0.08	.38	33.445	1/192	< .0001
				Average *T*_max_ (°C)	0.43	.09	0.309	1/9	.592
				Average precipitation (mm)	0.23	.08	0.146	1/9	.7113
				Soil texture (PC1)	0.37	.14	0.571	1/9	.469
				Time in chill coma (min)	−0.005	−.07	0.147	1/9	.7101
**c**	.4	28	Standard deviation in CT_max_	Sex	0.48	.55	0.531	1/15	.4774
				Average body mass (mg)	0.02	.59	1.988	1/12	.1836
				**Average *T***_**max**_ **(°C)**	−0.72	−.69	10.79	1/8	.0106
				Average precipitation (mm)	−0.06	−.1	0.108	1/8	.7505
				Soil texture (PC1)	0.12	.23	0.866	1/8	.3788
				Time in chill coma (min)	0.001	.04	0.028	1/8	.8716

*Note*: Average *T*_max_ is the average maximum daily temperature for the month of July (2019) collected at the site level (*n* = 14). Models include the adjusted *R* ^2^, coefficient (coef.), and standardized coefficient (*β*_std_). Predictors with *p* < .05 are bolded.

### 3.3 Effects of relative parasite intensity on CT_max_

**FIGURE 4:**
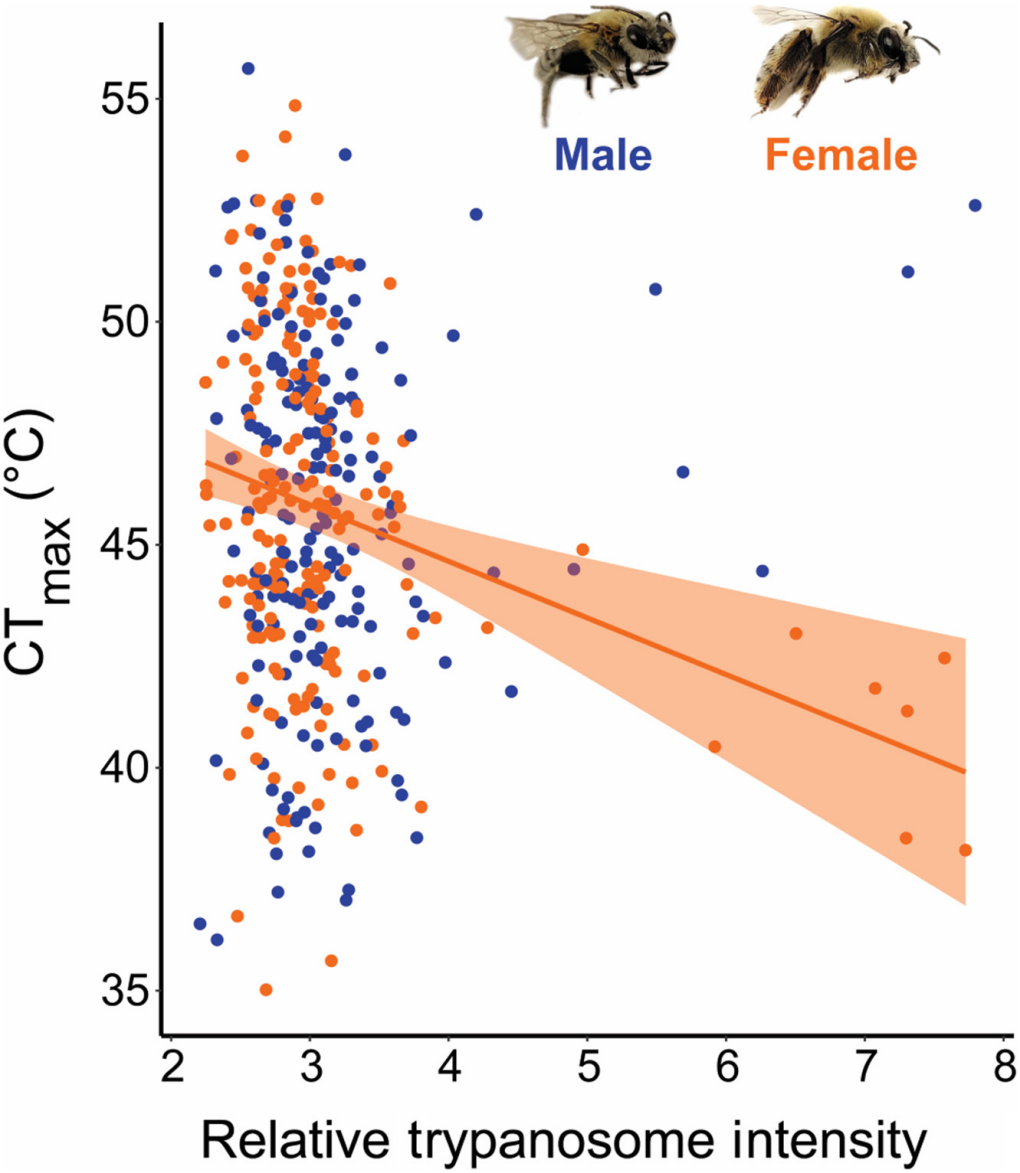
The effect of relative trypanosome intensity on *Xenoglossa pruinosa* CT_max_. Relative trypanosome intensity (log_10_(2^−ΔΔCT^)) (40 ng DNA per reaction) negatively correlates with female (orange; *y* = −0.6*x* + 47.63; 95% CI = (−1.12, −0.09); *n* = 198) but not male (blue; *n* = 176) CT_max_. The shaded region around the regression line represents the 95% confidence interval.

We detected trypanosomes in 89% of samples, *Vairimorpha apis* in 100% of samples, and *Spiroplasma apis* in 39% of samples. We found that trypanosomes were the only parasites to impact CT_max_ (Table 2 and Table S2), and that the effect was depended on sex (Table S2k). Female *X*. *pruinosa* CT_max_ was negatively correlated with trypanosome intensity (coef. = −1.08, *p* = .001), while males showed no relationship (*p* = .33) (Figure 4; Table 2d,e). In contrast, we found no effect of *S*. *apis* (mollicute bacteria; *p* = .26) or *V*. *apis* (microsporidian; *p* = .15) intensity on *X*. *pruinosa* CT_max_ (Figure S4; Table 2f–i and Table S2).

**TABLE 2:** Analysis of Variance (ANOVA) reporting Type II Wald *F* tests for linear mixed models (LMMs) including biotic (parasite intensity) predictors.

Mod.	*R* ^2^ (adj.)	*n*	Response	Predictor	Coef.	*β*_std_	*F* statistic	*df*	*p*‐Value
**d**	.1	198	Female CT_max_	**Body mass (mg)**	0.04	.22	9.504	1/190	.0024
				**Trypanosome intensity**	−1.08	−.23	11.204	1/192	.001
				Time in chill coma (min)	−0.003	−.06	0.299	1/12	.5947
**e**	.15	176	Male CT_max_	**Body mass (mg)**	0.34	.06	30.942	1/172	<.0001
				Trypanosome intensity	0.07	.39	0.934	1/168	.3352
				Time in chill coma (min)	−0.002	−.03	0.044	1/12	.837
**f**	.04	77	Female CT_max_	Body mass (mg)	0.03	.18	2.188	1/72	.1434
				*Spiroplasma apis Intensity*	−0.24	−.06	0.202	1/52	.6551
				Time in chill coma (min)	−0.01	−.09	0.476	1/9	.5074
**g**	.18	84	Male CT_max_	**Body mass (mg)**	0.10	.41	14.67	1/75	.0003
				*Spiroplasma apis intensity*	0.51	.18	1.661	1/27	.2083
				Time in chill coma (min)	0.003	.04	0.094	1/9	.7656
**h**	.06	220	Female CT_max_	**Body mass (mg)**	0.04	.24	11.835	1/213	.0007
				*Vairimorpha apis intensity*	0.85	.09	1.897	1/209	.1699
				Time in chill coma (min)	−0.004	−.07	0.354	1/12	.563
**i**	.15	198	Male CT_max_	**Body mass (mg)**	0.08	.39	34.809	1/194	<.0001
				*Vairimorpha apis intensity*	0.49	.04	0.627	1/185	.4296
				Time in chill coma (min)	−0.003	−.04	0.086	1/12	.775

*Note*: Models include the adjusted *R* ^2^, coefficient (coef.), and standardized coefficient (*β*_std_). Predictors with *p* < .05 are bolded.

## 4 DISCUSSION

When evaluating the thermal tolerance of ectotherms, it is essential to understand what abiotic and biotic conditions explain the variation in thermal tolerance among individuals in natural populations. In our study, we find evidence that body size, environmental temperature, and parasite infection have context‐ and sex‐dependent effects on heat tolerance in the solitary bee *X*. *pruinosa*. We find that although both sexes showed a positive correlation between heat tolerance and size, male *X*. *pruinosa* had a greater change in their CT_max_ per unit body mass than females, suggesting that there may be another biological trait influencing the impact of body mass on heat tolerance that differs between the sexes. While we did not find that extreme temperature (average daily *T*_max_), precipitation, or soil texture predicted mean CT_max_, our results show a strong negative relationship between the overall standard deviation in CT_max_ within populations and *T*_max_. This negative relationship between *T*_max_ and variation in heat tolerance may suggest that that extreme temperature is filtering out individuals with high and low CT_max_. Lastly, relative trypanosome intensity negatively impacted female heat tolerance, though this relationship appears driven by few individuals with relatively higher trypanosome titers. This suggests there is a threshold at which trypanosome infection impacts heat tolerance. Despite known negative impacts of the other two parasites *S*. *apis* (mollicute bacteria) and *V*. *apis* (microsporidian) on bee fitness (McArt et al., 2014), we found that they did not impact *X*. *pruinosa* heat tolerance.

Our study contradicts most current thermal tolerance work in bee systems that suggest that heat tolerance is not associated with body size (Burdine & McCluney, 2019; Gonzalez et al., 2020; Gonzalez, Oyen, Aguilar, et al., 2022; Gonzalez, Oyen, Ávila, & Ospina, 2022; Hamblin et al., 2017; Oyen & Dillon, 2018). Instead, our findings are more similar to several other studies of bees (Oyen et al., 2016), ants (Baudier et al., 2015; Baudier & O'Donnell, 2018; Cerdá et al., 1997; Ribeiro et al., 2012; Roeder et al., 2021), and other terrestrial ectotherms (González‐del‐Pliego et al., 2020; Janowiecki et al., 2020) that find larger individuals are more heat tolerant. These latter works attribute this relationship largely to water retention during CT_max_ experiments. We did not provide *X*. *pruinosa* individuals with any source of added humidity, or the opportunity to replenish fluids during CT_max_ trials, and used a slow temperature ramp rate (+0.25°C min^−1^) which can increase time under heat stress. Likewise, we did not know if *X*. *pruinosa* individuals had collected nectar in the field prior to capture. So, we posit that larger *X*. *pruinosa* may have performed better in our CT_max_ trials due to increased water retention. To our knowledge, our finding that the strength of this relationship can be sex‐specific is not reported among bees; however, sex differences in thermal tolerance traits (Laidlaw et al., 2020; Madeira et al., 2012; Missionário et al., 2022; Mitchell & Hoffmann, 2010), plasticity (Pottier et al., 2021), or in the impacts of the environment on thermal tolerance (Preston & Johnson, 2022) have been reported across several other taxonomic groups. Generally, studies that thoroughly consider potential sex differences are rare or do not find differences (Du et al., 2000; Gonzalez, Oyen, Aguilar, et al., 2022; González‐del‐Pliego et al., 2020). We posit that the greater relationship we see between male heat tolerance and body mass in our study is due to their smaller sizes overall, as males (average mass = 81 mg) were roughly 40% smaller than females (average mass = 121 mg). Thus, small male *X*. *pruinosa* may have been more vulnerable to water loss during our experiments than females, though this is not reflected as a sex difference in their overall CT_max_. We acknowledge that there could be other sex‐specific biological traits that we did not measure in this study that could better explain the sex difference we see here, such as differences in energy allocation to reproduction or fat storage. Overall, we predict that water retention is important for the relationship between body size and heat tolerance for terrestrial ectotherms, and may explain inconsistencies in the relevance of body size for heat tolerance among different taxa. Further, intraspecific differences in heat tolerance, such as among sexes of a species, may be explained by differences in size. We urge that more studies consider sex differences when evaluating species' thermal tolerances.

We found evidence that terrestrial ectotherms are limited in their ability to adapt or acclimate their heat tolerance. None of the environmental variables we considered, including average daily *T*_max_, precipitation, or soil texture, predicted *X*. *pruinosa* CT_max_, providing further support that heat tolerance is constrained (Brett, 1956). We show preliminary evidence that *X*. *pruinosa* persisting at sites with high daily *T*_max_ are experiencing heat stress. At these sites, populations are converging at similar heat tolerances; however, the variation in CT_max_ is dramatically reduced. This reduced variation in CT_max_ within populations may indicate that individuals experience prolonged heat stress at these sites, and so those with lower heat tolerances are excluded from occupying these habitats long‐term (i.e., temperature is acting as an environmental filter), and individuals that persist at these sites cannot maintain high heat tolerances due to stress (exposure to heat stress reduces heat tolerance, see Rezende et al., 2011; Sinclair et al., 2016). Youngsteadt et al. (2023) similarly found that urban ants were not acclimating their heat tolerance at warmer sites, and regularly occupied spaces hotter than their thermal preferences when foraging. We do not know the thermal preferences of *X*. *pruinosa*. However, this finding may suggest that behavioral buffering is not sufficient to protect terrestrial ectotherms from heat stress and its potential sublethal effects, including reduced fertility (Walsh et al., 2019; Zeh et al., 2012). We suggest that future studies measure the fitness effects of prolonged and acute exposure to heat stress for terrestrial ectotherms, and consider evaluating thermal preferences in addition to thermal tolerance.

Our study suggests that the effect of parasite infection on thermal tolerance is threshold‐dependent and can vary between sexes. We found that the heat tolerance of females, but not males, was negatively correlated with relative trypanosome intensity. However, we acknowledge that there is wide variation in heat tolerance at low infection levels, which may represent only trypanosome detection and not true infection, and that this trend appears driven by few relatively highly infected individuals. Pathogen infection has been found to reduce thermal tolerance for some ectotherms (Bates et al., 2011; Greenspan et al., 2017; Hector et al., 2019; Sherman, 2008); however, few studies have characterized sex differences in the impact of infection on thermal tolerance (see Laidlaw et al., 2020). Despite overall greater thermal resistance in female *Daphnia* compared to males, Laidlaw et al. (2020) found that female *Daphnia* infected with the bacteria *Pastueria ramosa* had significantly reduced heat tolerance, thereby negating any thermal tolerance advantage. This impact of infection stress on female thermal tolerance is particularly concerning given that population growth depends on female fecundity, and in our system, females are typically already less abundant than males (Cane et al., 2011). Additionally, *Crithidia bombi* (i.e., trypanosome) infection in bumble bees has been demonstrated to change energy allocation patterns in females and impact their reproduction (Brown et al., 2000; Goulson et al., 2018); however, we do not know if this parasite group has similar direct effects on *X*. *pruinosa* reproductive fitness. It is also important to acknowledge that there may be abiotic or biotic factors such as pesticide exposure (Op de Beeck et al., 2017) or reduced resource availability (Huey & Kingsolver, 2019), that may amplify the impacts of pathogen stress, which may partially contribute to the sex‐specific response we see. Thus, quantifying multiple stressors that individuals may be experiencing at small spatial scales may be informative in explaining the variation in impacts of infection on heat tolerance within a species. We suggest that future studies investigate the relationship between trypanosome infection and heat tolerance with controlled inoculation experiments to ensure normally distributed infections and to limit other sources of stress. Additionally, future studies should explicitly assess pathogen replication within the host to confirm trypanosome infection.

Overall, our study contributes to growing evidence that adaptation or acclimation of heat tolerance to local climate conditions is constrained for terrestrial ectotherms. However, we did not characterize the microclimates experienced by individuals within the same sites, which may vary greatly (Roeder et al., 2021) and help explain high within‐population variation in heat tolerance. We find that size and infection status may explain variation in CT_max_, and that CT_max_ is less variable at sites that reach higher *T*_max_. We found significant sex differences in the effects of body mass and infection status on *X*. *pruinosa* heat tolerance despite no difference in mean CT_max_ between the sexes, exemplifying the need for sex to be considered in future studies of thermal tolerance in natural populations. While we only found that one parasite group reduced *X*. *pruinosa* heat tolerance, we suspect that our study underestimates the impact of trypanosome infection because we collected individuals early in the adult season, but trypanosome intensity in *X*. *pruinosa* has been demonstrated to increase overtime (unpublished data; LJ Jones). More generally, the impacts of infection on thermal tolerance are largely understudied across taxa (Hector et al., 2021), so we encourage more studies to consider these effects. It is also important to acknowledge that there is evidence of facultative endothermy in some insects (Heinrich, 1972; Kovac & Stabentheiner, 2011; May, 1979; Roberts & Harrison, 1998; Stone, 1993). We suspect that *X*. *pruinosa* may be similar to other midsize bees, such as *Apis mellifera* (Heinrich, 1996), that can maintain high thoracic temperatures via internal heat‐exchange mechanisms for particular tasks like flight (unpublished data; RJ Schilder). However, the capacity for endothermy in *X*. *pruinosa* and many other bee species has not been characterized, despite its important implications for thermal tolerance. We suggest that future studies investigate the endothermic abilities of a wider breadth of bee species. Lastly, given that small‐bodied invertebrates are limited in their acclimation or adaptation capacity in heat tolerance, it is critical to identify the populations that are at risk under future climate regimes. We suggest that future investigations assess the thermal tolerances of populations across a species' distribution to identify those that are most vulnerable to local extinction.

## AUTHOR CONTRIBUTIONS

**Laura J. Jones:** Conceptualization (lead); data curation (lead); formal analysis (lead); funding acquisition (equal); investigation (lead); methodology (lead); visualization (lead); writing – original draft (lead); writing – review and editing (equal). **Douglas A. Miller:** Formal analysis (supporting); methodology (supporting); writing – review and editing (equal). **Rudolf J. Schilder:** Conceptualization (supporting); formal analysis (supporting); funding acquisition (equal); methodology (supporting); project administration (equal); resources (equal); software (equal); supervision (equal); validation (equal); writing – review and editing (equal). **Margarita M. López‐Uribe:** Conceptualization (supporting); formal analysis (supporting); funding acquisition (lead); methodology (equal); project administration (lead); resources (equal); software (equal); supervision (equal); validation (equal); writing – review and editing (equal).

## FUNDING INFORMATION

This research was supported by the Pennsylvania Department of Agriculture, Grant number C940000082, and the USDA‐NIFA‐AFRI Pollinator Health Program, Project 2022‐67013‐36274. LJJ was supported by the NSF GRFP Grant Number DGE1255832. Any opinions, findings, and conclusions or recommendations expressed in this material are those of the author(s) and do not necessarily reflect the views of the National Science Foundation. RJS was funded through the USDA NIFA/Hatch Appropriations under project PEN04770. MML‐U was funded through the USDA NIFA Appropriations under Projects PEN04716 and PEN04620.

## CONFLICT OF INTEREST STATEMENT

The authors declare no conflicts of interest.

## Supporting information


Data S1.

## Data Availability

Data and R script are available in the Dryad Digital Repository and can be accessed at https://doi.org/10.5061/dryad.hhmgqnkp9.
