# Supplementary material for: Body mass, temperature, and pathogen intensity differentially affect critical thermal maxima and their population‐level variation in a solitary bee
Source: Ecol Evol. 2024 Feb 15;14(2):e10945. doi: 10.1002/ece3.10945 (PMC10867875; doi:10.1002/ece3.10945)
Supplement: Supplementary file 1 — Data S1. [file ECE3-14-e10945-s001.zip › ece310945-sup-0001-AppendixS1.docx]

**APPENDIX**

**
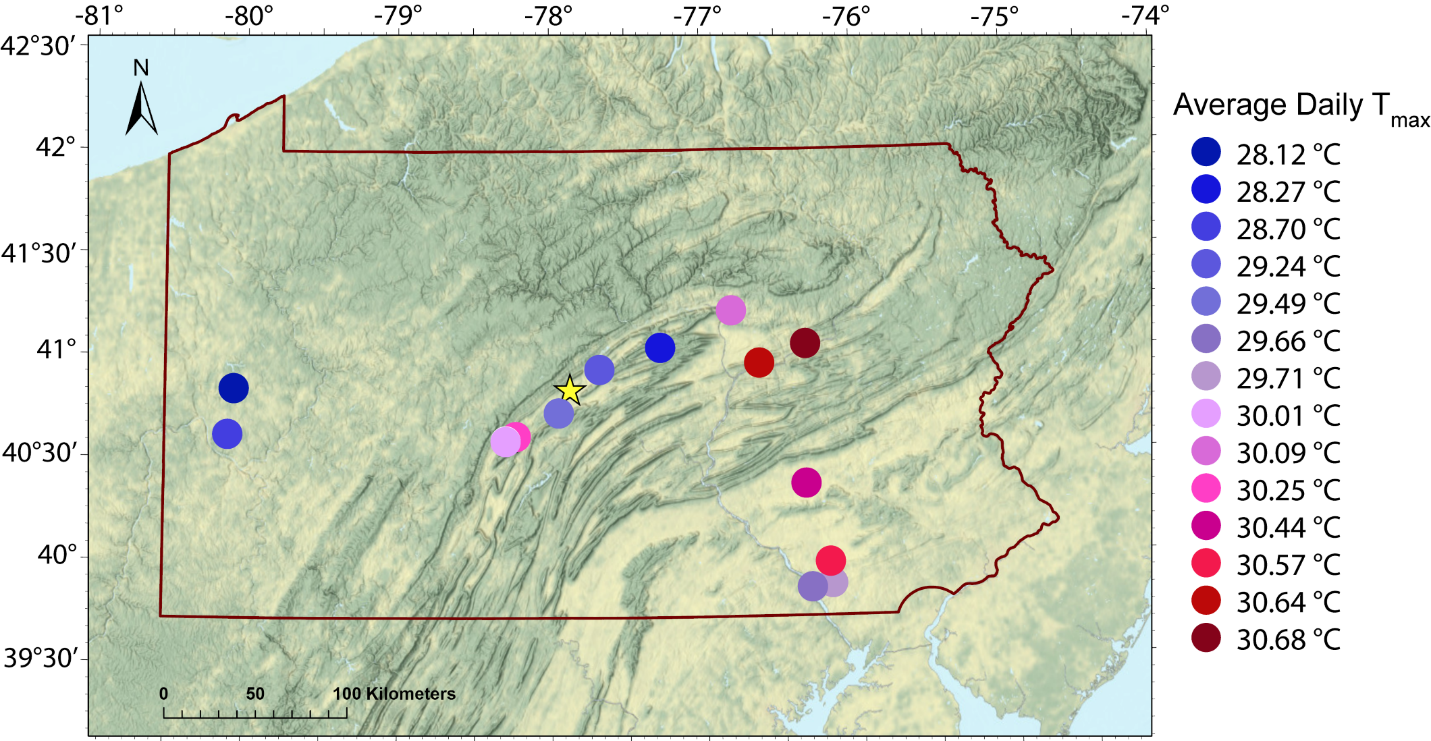
**

**FIGURE S1** *Xenoglossa pruinosa* were collected from fourteen *Cucurbita* farms (circles) in Pennsylvania (USA) in 2019. Colors represent the average daily maximum temperature estimated for the month of July in 2019 from PestWatch at each site (dark blue → dark red; see the key for the true value). Yellow star indicates The Pennsylvania University, Centre Co. PA (USA) where the CT_max_ assays took place. Axes indicate latitude (y-axis) and longitude (x-axis). The base topographic layer is copyright 2021 General Bathymetric Chart of the Oceans base map with National Oceanic and Atmospheric Administration’s National Centers for Environmental Information visualization.


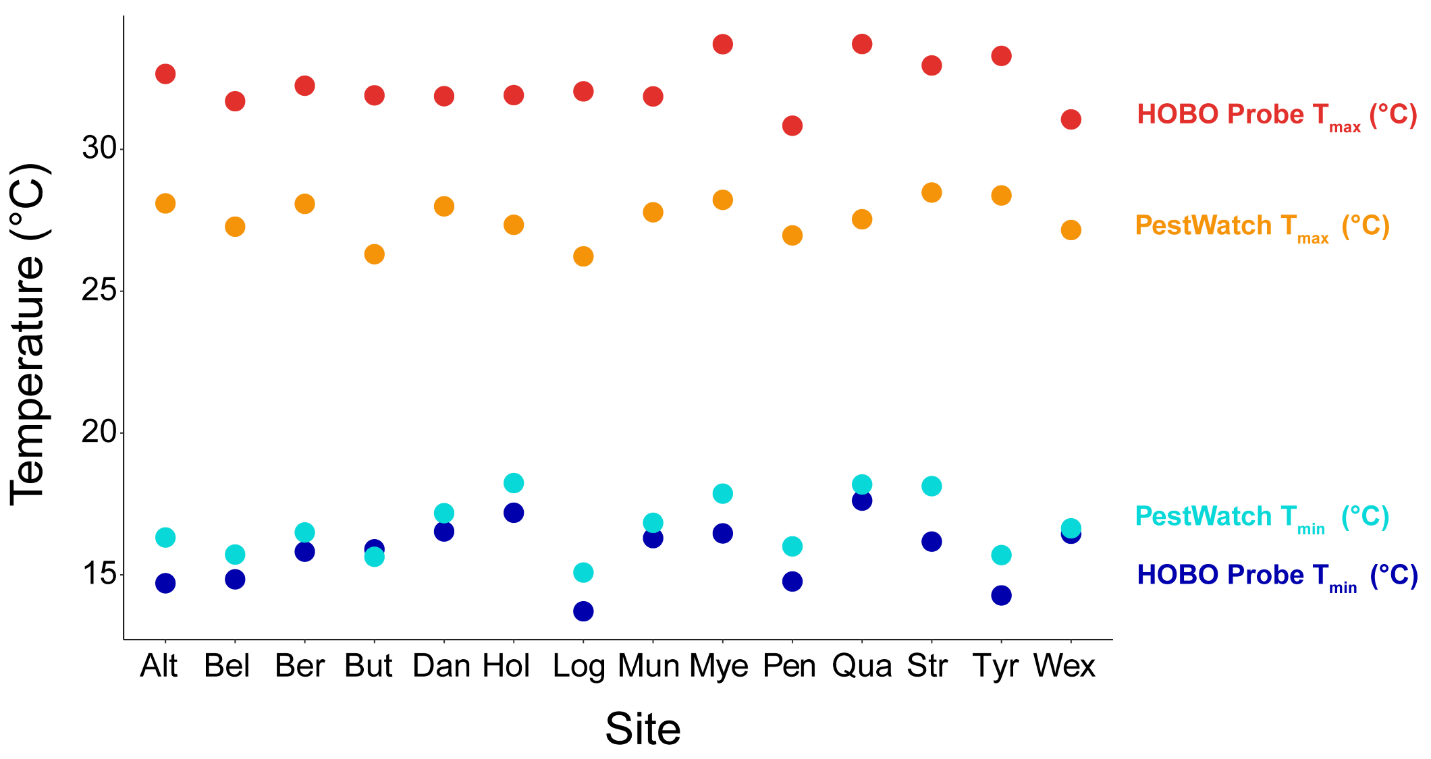


**FIGURE S2** The average daily maximum and minimum temperatures for the month of August (2019) recorded at each site (14 *Cucurbita* fields, PA (USA)). Points show average daily minimum (dark blue) and maximum (red) temperatures recorded from HOBO temperature probes in the field at each site compared to average daily minimum (turquoise) and maximum (orange) temperatures estimated by the model PestWatch at a spatial resolution of 4 km at each site. For analyses, we used average daily maximum temperatures collected during the month of July by PestWatch, which is not depicted here (see Figure 3 in the main text).


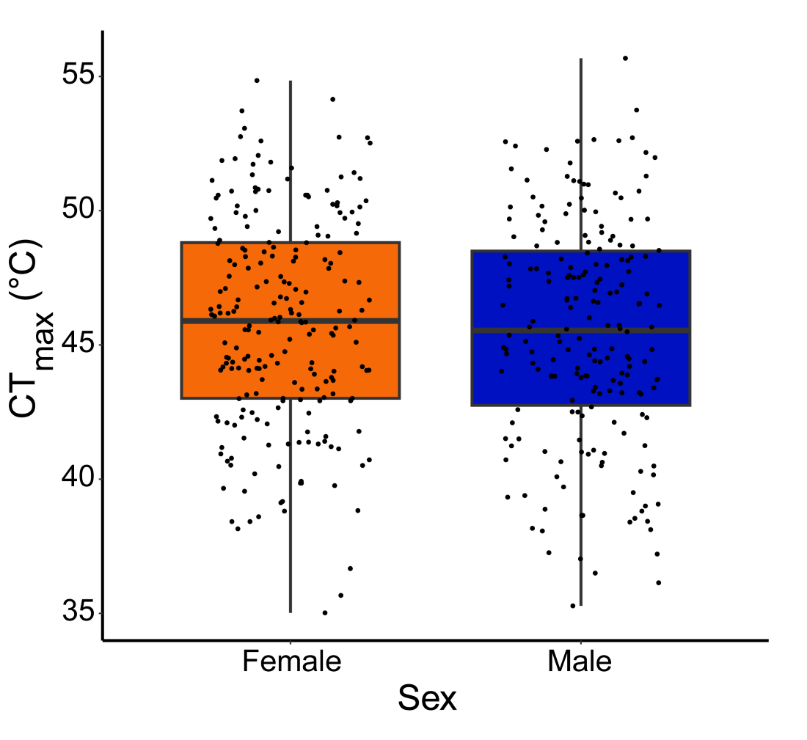


**FIGURE S3** Boxplots comparing the CT_max_ between *Xenoglossa pruinosa* sexes. The average CT_max_ for females was 45.82 ± 3.93 °C and the average CT_max_ for males was 45.47 ± 4.14 °C. There is no difference in CT_max_ between *X. pruinosa* sexes (Tukey HSD, p > 0.05).


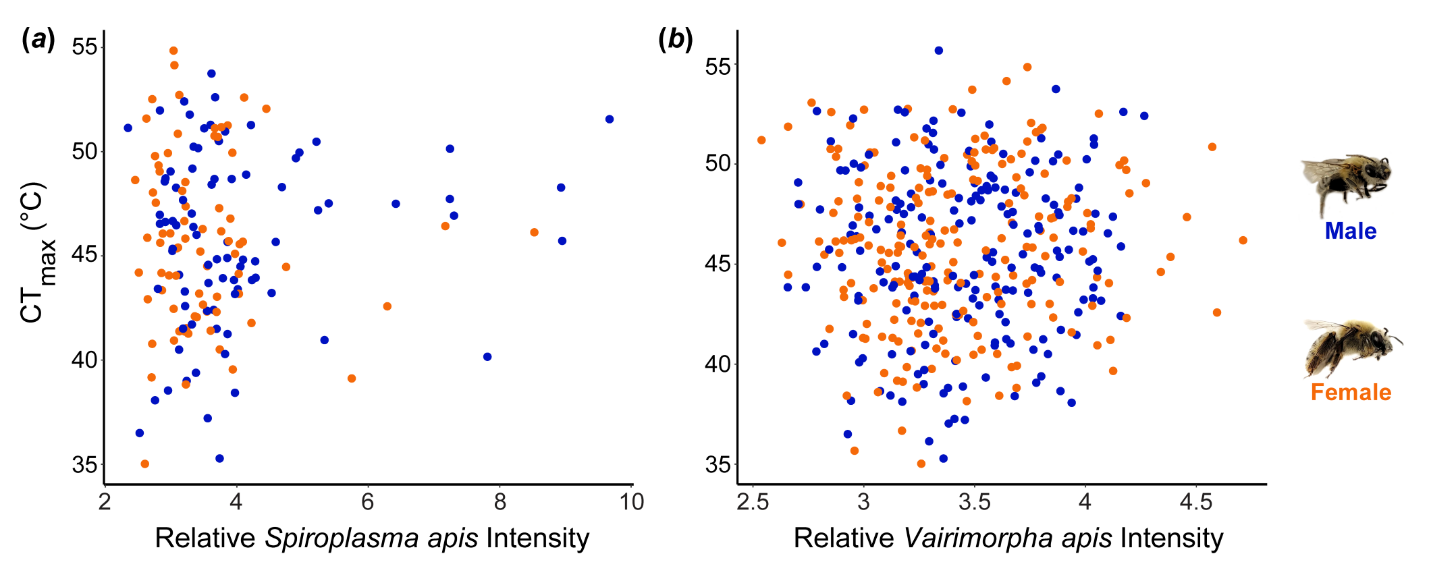


**FIGURE S4** The relative **(a)** *Spiroplasma apis* intensity (log_10_(2^−ΔΔCT^)) (detected in 77 females and 84 males) and **(b)** *Vairimorpha apis* intensity (detected in 220 females and 198 males) compared to *Xenoglossa pruinosa* CT_max_. All qPCR reactions included 40 ng of DNA.

**TABLE S1** Primers used for qPCR detection and relative quantification of trypanosomes, *Spiroplasma apis*, *Vairimorpha apis*, and the reference gene 28S. Primer efficiency (primer eff.) and amplification factor (amp. factor) are indicated.

| **Target** | **Gene** | **Forward Seq. (5′-3′)** | **Reverse Seq. (5′-3′)** | **Size (bp)** | **Primer Eff. Equation** | **Primer Eff. (%)** | **Amp. Factor** | **Dilution Factor** | **Reference** |
| --- | --- | --- | --- | --- | --- | --- | --- | --- | --- |
| Trypanosomes | 18S rRNA | GGCCAC CCACGG GAATAT | CAAAGC TTTCGC GTGAAG AAA | 56 | y = -0.338x + 7.41;  R^2^ = 0.997 | 117.8 | 2.18 | 10 | Ulrich et al. (2011) |
| *Spiroplasma apis* | 16S rRNA | AATGCC AGAAGC ACGTAT CC | GAACGA GATATA CTCATA AGCTGT TACAC | 190 | y = -0.313x + 5.85;  R^2^ = 0.999 | 105.6 | 2.06 | 10 | Schwarz et al. (2014) |
| *Vairimorpha apis* | 16S rRNA | CAATAT TTTATT GTTCTG CGAGG | AAAGTC TATTGT ATTGCG CGTGCT | 208 | y = -0.36x + 11.6;  R^2^ = 0.806 | 129.1 | 2.29 | 5 | Natsopoulou et al. (2017) |
| Apocrita | 28S rRNA | TGGTTC CCTCCG AAGTTT CCCTCAG | GCAAGC CAGAGA TCTCAC CCATTTA | 141 | NA | NA | NA | NA | Alburaki et al. (2018) |

**TABLE S2** Analysis of Variance (ANOVA) reporting Type II Wald F tests for linear mixed models (LMMs) and Type II sum of squares tests for linear models with sexes combined in analyses. Each model output includes the adjusted R^2^ and standardized coefficient (β_std_). Model residuals are normally distributed.

| **Mod.** | **R^2^ (adj.)** | **n** | **Response** | **Random Effect** | **Predictor** | **Coef.** | **β_std_** | **F Statistic** | **DF** | **P Value** |
| --- | --- | --- | --- | --- | --- | --- | --- | --- | --- | --- |
| **j** | 0.15 | 418 | CTmax | Site | **Sex** | -4.33 | 0.46 | 8.986 | 1/410 | 0.0029 |
|  |  |  |  |  | **Body Mass (mg)** | 0.03 | 0.21 | 34.895 | 1/403 | < 0.0001 |
|  |  |  |  |  | Average Tmax (°C) | 0.32 | 0.07 | 0.333 | 1/9 | 0.5783 |
|  |  |  |  |  | Average Precipitation (mm) | 0.32 | 0.12 | 0.547 | 1/9 | 0.4783 |
|  |  |  |  |  | Soil Texture (PC1) | 0.10 | 0.04 | 0.088 | 1/9 | 0.7729 |
|  |  |  |  |  | Time in Chill Coma (min) | -0.01 | -0.11 | 0.567 | 1/9 | 0.4708 |
|  |  |  |  |  | **Sex:Body Mass (mg)** | 0.06 | 0.45 | 15.153 | 1/404 | 0.0001 |
| **k** | 0.12 | 374 | CTmax | Site | **Sex** | -3.71 | 0.5 | 10.853 | 1/368 | 0.0011 |
|  |  |  |  |  | **Body Mass (mg)** | 0.03 | 0.22 | 32.416 | 1/362 | < 0.0001 |
|  |  |  |  |  | Trypanosome Intensity | -0.43 | -0.08 | 3.052 | 1/368 | 0.0815 |
|  |  |  |  |  | Time in Chill Coma (min) | -0.003 | -0.05 | 0.191 | 1/12 | 0.6698 |
|  |  |  |  |  | **Sex:Body Mass (mg)** | 0.06 | 0.42 | 11.727 | 1/360 | 0.0007 |
| **l** | 0.09 | 161 | CTmax | None | **Body Mass (mg)** | 0.03 | 0.21 | 14.577 | 1/155 | 0.0002 |
|  |  |  |  |  | **Sex** | -5.05 | 0.6 | 6.0976 | 1/155 | 0.0146 |
|  |  |  |  |  | *Spiroplasma apis Intensity* | 0.28 | 0.09 | 1.2589 | 1/155 | 0.2636 |
|  |  |  |  |  | Time in Chill Coma (min) | -0.002 | -0.03 | 0.1363 | 1/155 | 0.7124 |
|  |  |  |  |  | **Sex:Body Mass (mg)** | 0.07 | 0.52 | 6.002 | 1/155 | 0.0154 |
| **m** | 0.13 | 418 | CTmax | Site | **Body Mass (mg)** | 0.03 | 0.23 | 37.8697 | 1/405 | < 0.0001 |
|  |  |  |  |  | **Sex** | -4.22 | 0.47 | 9.7774 | 1/412 | 0.0019 |
|  |  |  |  |  | *Vairimorpha apis Intensity* | 0.64 | 0.06 | 2.0971 | 1/402 | 0.1484 |
|  |  |  |  |  | Time in Chill Coma (min) | -0.003 | -0.05 | 0.2581 | 1/12 | 0.6207 |
|  |  |  |  |  | **Sex:Body Mass (mg)** | 0.06 | 0.45 | 14.8883 | 1/404 | 0.0001 |

**TABLE S3** Moran’s I test for spatial autocorrelation in CT_max_ and trypanosome, *Spiroplasma apis*, and *Vairimorpha apis* intensities among *Xenoglossa pruinosa* across sites. Expected Moran’s I under the null hypothesis is -0.0769. Site-level means for each variable were calculated with sexes pooled.

| **Variable** | **Moran's I** | **Standard Deviation** | **P Value** |
| --- | --- | --- | --- |
| Average CTmax | -0.055 | 0.145 | 0.878 |
| Trypanosomes Intensity | -0.037 | 0.136 | 0.766 |
| *Spiroplasma apis* Intensity | -0.110 | 0.069 | 0.635 |
| *Vairimorpha apis* Intensity | -0.197 | 0.137 | 0.381 |

**Extended Methods**

**Section 2.3: Environmental Conditions**

We collected hourly temperature data for the month of August at each site using two HOBO 8K Pendant temperature data loggers (UA-001-08). We placed one data logger at the edge of the field and the second data logger 11 meters into the field. We built shields to protect the HOBO data loggers from solar radiation following the design described in Terando et al. (2017).

**Section 2.5: Statistical Analyses**

We used the packages dplyr (v.1.0.7) (Wickham et al., 2023) and tidyr (v.1.2.1) (Wickham & Girlich 2022) for data manipulation. Figures were created using packages ggplot2 (v.3.3.2) (Wickham, 2016) and ggpubr (v.0.4.0) (Kassambara, 2020). We checked for correlation among our environmental predictors and selected the average maximum daily temperature estimated by PestWatch during the month of July as our temperature predictor, the average daily precipitation for the month of July as our precipitation predictor, and the soil texture principle component 1 as our soil texture predictor (functions ‘vif’ and ‘cor’, package cor (v.3.6.2)) (Becker et al., 1988). We identifieparadis

d two outliers in the distribution of CT_max_, two male *X. pruinosa* with CT_max_ < 30 °C, which we believe were in poor condition (functions ‘hist’ and ‘qqnorm’, package stats (v.3.6.2)) (Becker et al., 1988). We ran models both with and without these two individuals included and found no difference in significant effects. Here we present models with these outliers removed as they provide more conservative results and we believe the data without these outliers reflect a more accurate distribution of CT_max_. For model c, the average body mass per *X. pruinosa* sex per site was used because the response (standard deviation of CT_max_) is a site-level variable. We compared model fit with sex and body mass included as interacting effects or as separate fixed effects in all models that included both predictors by comparing Akaike information criterion scores using Analysis of Variance (ANOVA), and found that models j-m (Table S2) performed better with this interaction (‘anova’, car(v.3.0-10)) (Fox and Weisburg, 2019). We confirmed that the residuals of all models were normally distributed (functions ‘qqnorm(resid)’ and ‘hist(resid)’, package stats (v.3.6.2)) (Becker et al., 1988). We did not include sample date as an effect in our models due to overfitting, and because we found that it was not correlated with CT_max_ (estimate = -0.038, F value = 0.83, p value = 0.36).

**Appendix References**

Alburaki, M., Chen, D., Skinner, J., Meikle, W., Tarpy, D., Adamczyk, J., & Stewart, S. (2018). Honey bee survival and pathogen prevalence: From the perspective of landscape and exposure to pesticides. *Insects*, *9*(2), 65. https://doi.org/10.3390/insects9020065

Becker, R. A., Chambers, J. M., & Wilks, A. R. (1988). *The New S Language*. Wadsworth & Brooks/Cole. https://www.rdocumentation.org/packages/stats/versions/3.6.2/topics/qqnorm

Kassambara, A. (2020). “ggplot2” Based Publication Ready Plots. *CRAN*. https://rpkgs.datanovia.com/ggpubr/

Natsopoulou, M. E., McMahon, D. P., Doublet, V., Frey, E., Rosenkranz, P., & Paxton, R. J. (2017). The virulent, emerging genotype B of Deformed wing virus is closely linked to overwinter honeybee worker loss. *Scientific Reports,* *7*(1), 1–9. https://doi.org/10.1038/s41598-017-05596-3

Schwarz, R. S., Teixeira, É. W., Tauber, J. P., Birke, J. M., Martins, M. F., Fonseca, I., & Evans, J. D. (2014). Honey bee colonies act as reservoirs for two *Spiroplasma* facultative symbionts and incur complex, multiyear infection dynamics. *MicrobiologyOpen*, *3*(3), 341–355. https://doi.org/10.1002/MBO3.172

Terando, A. J., Youngsteadt, E., Meineke, E. K., & Prado, S. G. (2017). Ad hoc instrumentation methods in ecological studies produce highly biased temperature measurements. *Ecology and Evolution*, *7*(23), 9890–9904. https://doi.org/10.1002/ECE3.3499

Ulrich, Y., Sadd, B. M., & Schmid-Hempel, P. (2011). Strain filtering and transmission of a mixed infection in a social insect. *Journal of Evolutionary Biology*, *24*(2), 354–362. https://doi.org/10.1111/J.1420-9101.2010.02172.X/FORMAT/PDF

Wickham, H. (2016). ggplot2: Elegant graphics for data analysis. *Springer-Verlag*. https://ggplot2.tidyverse.org

Wickham, H., François, R., Henry, L., Müller, K., & Vaughan, D. (2023). *dplyr: A grammar of data manipulation*. https://dplyr.tidyverse.org, https://github.com/tidyverse/dplyr

Wickham Hadley, & Girlich Maximilian. (2022). tidyr: tidy messy data. *Https://Tidyr.Tidyverse.Org, Https://Github.Com/Tidyverse/Tidyr*.
